# Yield estimation of high-density cotton fields using low-altitude UAV imaging and deep learning

**DOI:** 10.1186/s13007-022-00881-3

**Published:** 2022-04-27

**Authors:** Fei Li, Jingya Bai, Mengyun Zhang, Ruoyu Zhang

**Affiliations:** 1 College of Mechanical and Electrical Engineering, Shihezi University, Shihezi, 832000 Xinjiang People’s Republic of China; 2 Key Laboratory of Northwest Agricultural Equipment, Ministry of Agriculture, Shihezi, 832000 Xinjiang People’s Republic of China

**Keywords:** Yield estimation, Unmanned aerial vehicle, SegNet, Densely planted cotton

## Abstract

**Background:**

China has a unique cotton planting pattern. Cotton is densely planted in alternating wide and narrow rows to increase yield in Xinjiang, China, causing the difficulty in the accurate estimation of cotton yield using remote sensing in such field with branches occluded and overlapped.

**Results:**

In this study, unmanned aerial vehicle (UAV) imaging and deep convolutional neural networks (DCNN) were used to estimate densely planted cotton yield. Images of cotton fields were acquired by the UAV at an altitude of 5 m. Cotton bolls were manually harvested and weighed afterwards. Then, a modified DCNN model (CD-SegNet) was constructed for pixel-level segmentation of cotton boll images by reorganizing the encoder-decoder and adding dilated convolutions. Besides, linear regression analysis was employed to build up the relationship between cotton boll pixels ratio and cotton yield. Finally, the estimated yield for four cotton fields were verified by weighing harvested cotton. The results showed that CD-SegNet outperformed the other tested models, including SegNet, support vector machine (SVM), and random forest (RF). The average error in yield estimates of the cotton fields was as low as 6.2%.

**Conclusions:**

Overall, the estimation of densely planted cotton yields based on low-altitude UAV imaging is feasible. This study provides a methodological reference for cotton yield estimation in China.

## Background

Xinjiang is the most important cotton planting base in China and it occupies a pivotal position in the world's cotton industry. In 2020, China's total cotton output was 5.91 million tons, of which 87.3% (5.16 million tons) were produced in Xinjiang. Fast and reliable estimation of cotton yield prior to harvest is essential for crop management, cotton trade, and policy making. At present, farmers in Xinjiang widely adopt the dense planting pattern of “short-dense-early”. This pattern employs alternating wide (66 cm) and narrow (10 cm) rows, and the number of plants per hectare is between 240,000 and 270,000. Although this pattern has obvious advantages in withstanding natural disasters and increasing yield [1], the plant density is relatively high. Moreover, narrow rows of cotton plants are staggered and severely occluded, which poses certain difficulties for imaging-based yield estimation.

The traditional cotton yield estimation methods are laborious and inefficient, and cannot meet the needs of the rapidly developing cotton industry [2]. Within a cotton field, there may be spatial differences in yields, which may introduce large errors in the estimates. With the continuous development of space technology, crop yield estimation methods based on satellite remote sensing technology have been widely used [3–5]. Cotton yield can be accurately predicted by using yield estimation models constructed with remote sensing data as well as vegetation index [6]. However, satellite remote sensing images can be affected by temporal and spatial resolution as well as cloud cover, so they are usually not enough to accurately estimate crop yields on the field scale. In contrast, unmanned aerial vehicles (UAVs) have quickly become ideal tools for precise crop monitoring due to their flexibility and low-altitude flight capability [7, 8]. UAV-based low-altitude remote sensing platform can obtain high spatial–temporal resolution images free from atmospheric interference [9–11]. For example, Akash et al. [12] developed a machine learning framework for estimating cotton yield using multi-temporal remote sensing data collected with unmanned aerial systems (UASs) to obtained more reliable crop yield estimates. Stroppiana et al. [13] accurately estimated wheat and soybean yields using low-altitude UAV remote sensing.

Vegetation index is only suitable for estimating cotton yield in the mid-growth stage, and has limited performance in the mature stage. Due to the influence of boll opening and background objects such as branches and leaves in the later growth stages of cotton, obvious differences are always found in visual characteristics such as colour and morphology. However, cotton yield can be directly estimated by remote sensing images and background segmentation of deciduous cotton fields. For example, Huang et al. [14] used UAV images to estimate cotton yield based on cotton boll coverage and plant height. Feng et al. [15, 16] comprehensively evaluated the image characteristics at different growth stages of cotton when estimating yield, and it was found that plant height and cotton fiber index were important features for estimating cotton yield before harvest. Xu et al. [17] constructed a cotton yield estimation model based on UAV remote sensing data. However, in the above yield estimations, the density of cotton plants is lower than that in Xinjiang, China, and the interlacing between cotton plants is relatively inapparent. Xu et al. constructed a model to predict single boll weight of densely planted cotton by using high resolution UAV remote sensing data, and it was found that the pixels ratio of opening bolls exhibited a strong correlation with the single boll weight in the upper layer. However, no conclusion was given on the yield estimates of the plot [18]. In addition, the above researches acquired orthomosaic images of the entire cotton field. Generating these types of images is complicated and time-consuming. Many scholars have tried to develop various ground-based sensing systems. For example, a digital camera installed on a robotic platform was used to estimate the number of cotton bolls based on images acquired by the 3D sensor system, boll estimates, or lint obtained from point clouds [19]. With the calculation of the number of cotton bolls in the field, accurate cotton yield prediction can be achieved [20]. However, the high density of cotton makes the movement of ground sensing platform and image acquisition difficult, and affects the estimation accuracy. Therefore, using low altitude UAVs as ground-based sensing platforms to acquire images may be a better choice.

In addition, significant advances in data collection and computing in recent years have facilitated the rapid development of deep learning (DL). As a powerful feature learning algorithm, DL outperforms traditional feature extraction methods in many fields. Li et al. [21] used a full convolutional network (FCN) and interference region removal module to segment the remote sensing data of cotton in the field. Ma et al. [22] proposed the EarSegNet semantic segmentation method, which can achieve accurate segmentation of wheat ears from canopy images acquired during the flowering period. However, the images used in previous studies were all taken from fixed platforms, not UAVs.

In this study, a method based on DL and low-altitude UAV imaging was proposed to estimate the yield of densely planted cotton after defoliation. Low-altitude UAV imaging was used for image acquisition, and pixel-level semantic segmentation was then applied to raw UAV images to acquire cotton boll pixels ratio. Finally, a yield estimation model was constructed based on the pixels ratio of cotton bolls. The specific objectives were to: (1) efficiently and accurately segment cotton bolls from the images collected using UAVs during the defoliation period; (2) construct and verify the constructed yield estimation model based on a single image feature; and (3) evaluate the yield estimation accuracy for multiple cotton fields.

## Results

### Performance evaluation

In this study, four segmentation models were recombined using the designed compilation and decoding blocks, namely, Model 1, Model 2, Model 3, and Model 4. The trained models were used to test 800 images in the test set and the evaluation results of the selected segmentation approaches in terms of mIoU, Recall, Precision, and F1-Score were presented. Based on the results of test set (Table 1), Model 1 had the lowest accuracy, while its recall was the highest. Model 4 had the highest accuracy with average mIoU of 77.13%, recall of 84.71%, precision of 90.82%, and F1-Score of 87.93%. Under the same number of convolution blocks, the models performed better after dilated convolution was added. The results for the training and testing of other algorithms on the same image data are shown in Table 2. The results showed that the modified model outperformed the original SegNet model and traditional machine learning algorithms (support vector machine (SVM) and random forest (RF)) in cotton field image segmentation. This may be due to that deep learning model has a series of convolution structure that can extract additional features without manual design.

**Table 1:** Comparison of results of different encoder and decoder methods

Segmentation model	mIoU (%)	Recall (%)	Precision (%)	F1-score (%)
Model 1	74.63	88.36	81.35	84.45
Model 2	74.85	88.77	83.84	85.29
Model 3	73.48	84.52	86.61	85.35
Model 4	77.13	84.71	90.82	87.93

**Table 2:** Segmentation results comparing CD-SegNet with SegNet, SVM, and RF

Model	mIoU (%)	Recall (%)	Precision (%)	F1-score (%)
CD-SegNet	77.13	84.71	90.82	87.93
SegNet	74.52	81.36	89.71	85.47
SVM	64.27	78.28	73.42	75.58
RF	58.63	66.84	78.51	72.16

**Fig. 1:**
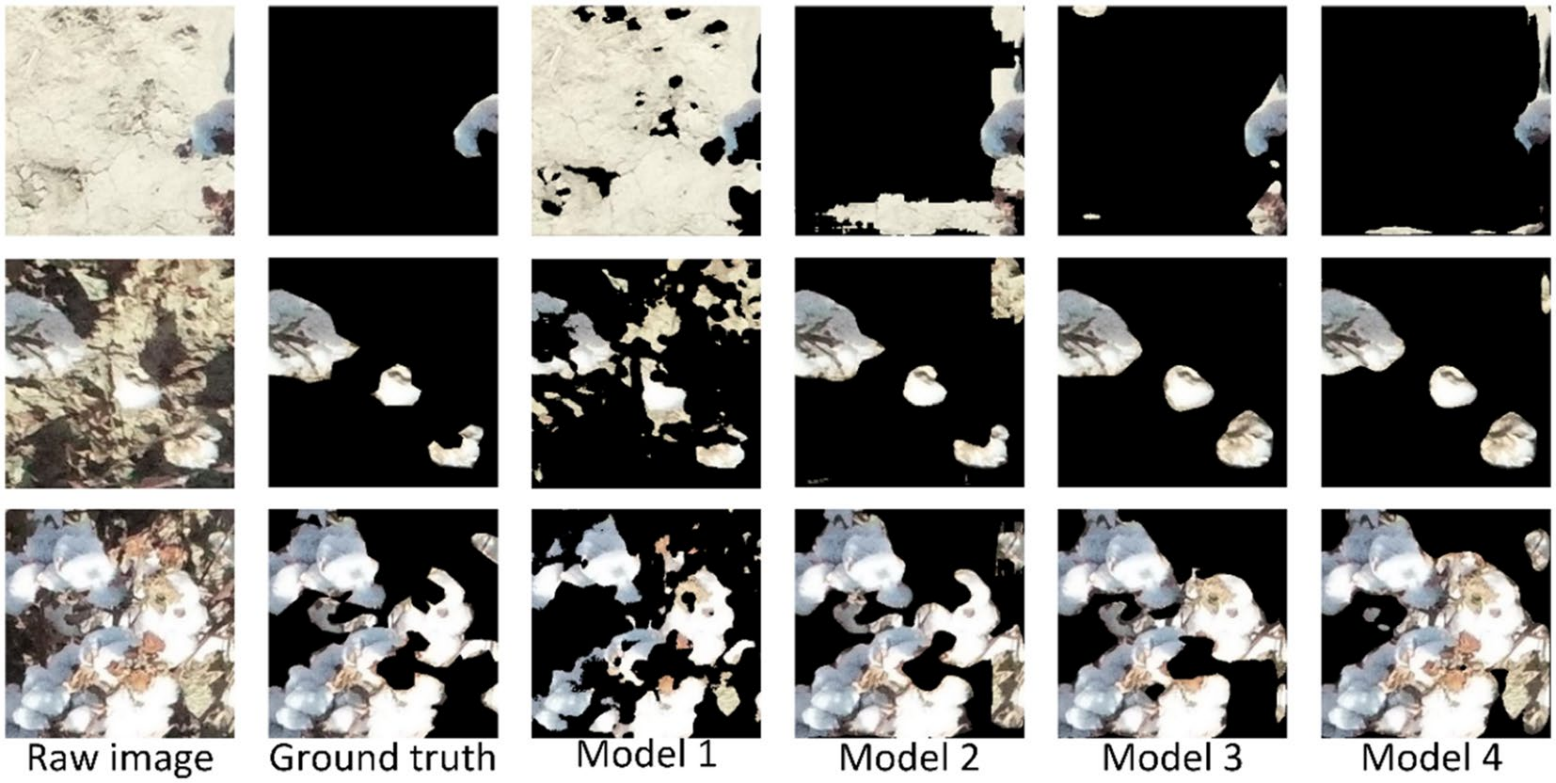
Segmentation results of complex background images with different models

Figure 1 shows the segmentation results of the above models. Model 1 had the worst segmentation, in particular, a large area of field was incorrectly segmented. Model 2 outperformed Model 1. However, there were still some errors in the segmentation at image edges. Both Model 3 and Model 4 showed better segmentation. In particular, Model 4 achieved better performance for boll segmentation under the reflective ground and occluded cotton leaves conditions. Thereby, it had a better segmentation logic. Therefore, Model 4 was selected as CD-SegNet.

### Sampled image segmentation

**Fig. 2:**
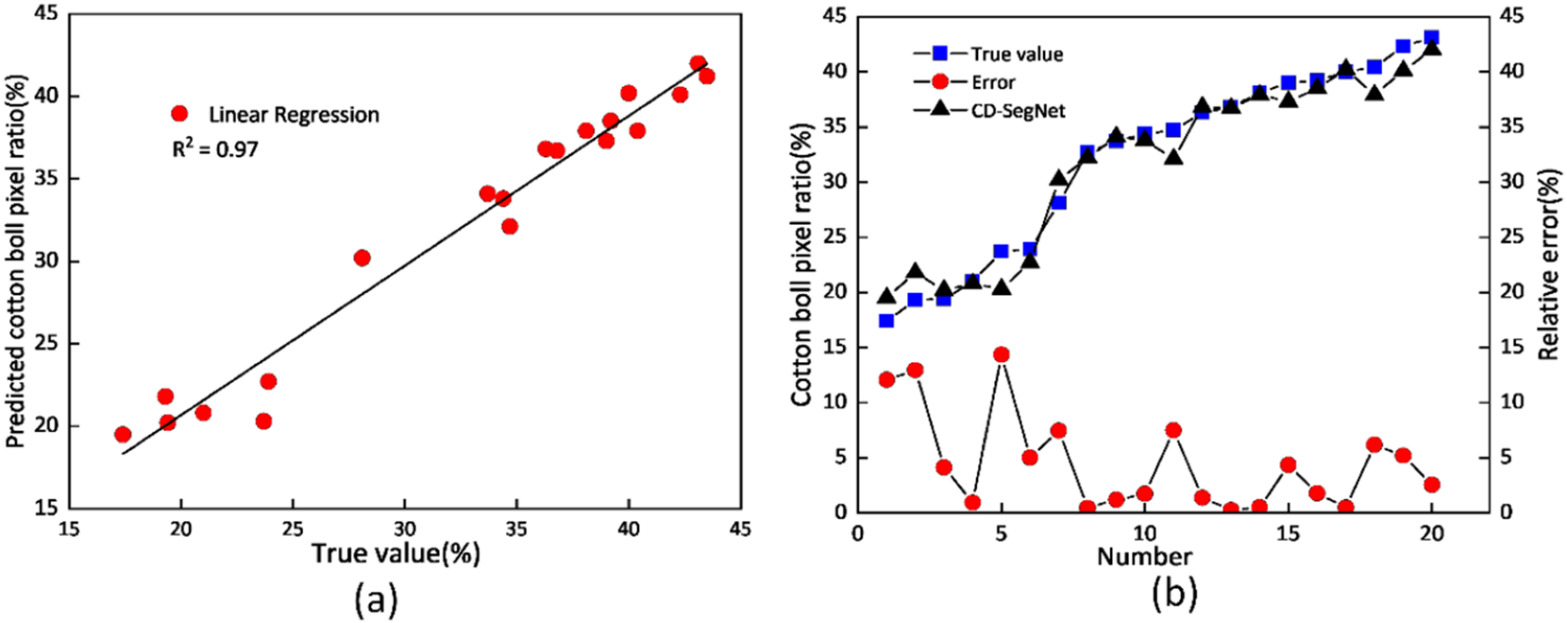
Comparison of CD-SegNet segmentation results with the measured area ratio of cotton bolls in the images. **a** Correlation between the measured data and the CD-SegNet segmentation results; **b** Relative error analysis

The CD-SegNet was used to segment 20 images to calculate the pixels ratio of cotton bolls. The results were then compared with the manually measured results. Figure 2a shows the correlation between the ground truth value and the CD-SegNet segmentation results. The coefficient of determination (R^2^) was 0.97. Figure 2b shows that the relative errors of boll pixels ratio obtained by using CD-SegNet for the image were in the range of 0.27–14.35%, and the average relative error was 4.77%. Figure 2b also shows that when cotton boll pixels ratio was less than 25%, the relative error was larger, and the ground truth value was less than the segmentation value; when the cotton boll pixels ratio was greater than 25%, the relative error decreased, and the ground truth value was greater than the segmentation value. By reviewing the segmented image, we found that this phenomenon was caused by the misalignment of the exposed ground and boll boundaries. Therefore, the CD-SegNet method can accurately segment the cotton boll pixels to calculate area ratio. However, in some cases, its performance may be limited by the light and background conditions.

### Yield estimation of cotton field

Regression relationship between cotton boll pixels ratio and measured yield was y = 38.6x + 34 (y: yield of the sample area (g/2.3 m^2^); x: cotton boll pixels ratio), with R^2^ of 0.91. Using the cotton boll pixels ratio calculated by CD-SegNet segmentation, the yield of each cotton field was estimated, and the estimates were compared with the measured yield. As shown in Table 3, the relative errors of the yield estimates were in the range of 0.67–10.5%, and increased with the measured yield. The UAV images obtained in this study were orthophoto images. In the vertical view, the lower cotton bolls may be obscured by the upper bolls, branches and leaves. In the same area, a higher yield means that more cotton bolls were obscured.

**Table 3:** Estimation of cotton yield in different fields using the area ratio of cotton bolls

Cotton field	Measured yield (kg ha^−1^)	Estimated yield (kg ha^−1^)	Difference between measured and estimated yield (kg ha^−1^)	Relative error of yield estimation (%)
1	5090	5124	34	0.67
2	6480	7158	678	10.5
3	5350	5116	234	4.4
4	5843	6391	548	9.4
Average error				6.2

## Discussion

In this research, the images were collected after the cotton was defoliated, which is different from the time in previous studies [6, 12, 17, 23, 24]. In cotton fields, not only the environment is unstructured and the illumination is changeable, but there are also mutual occlusion of cotton branches and clustering of cotton buds. These factors can complicate the background of the acquired images, which brings challenges to cotton boll pixel segmentation and yield estimation. In this study, a modified SegNet algorithm was proposed. The results showed that the proposed CD-SegNet model performed well for cotton boll pixel identification in dense planting mode (Fig. 2), and the relative errors lowered as the cotton boll pixels ratio increased. Furthermore, the yield prediction based on cotton boll pixels ratio was more accurate (average relative error of 6.2%) than conventional methods. The cotton boll pixels ratio of each cotton field was the average of 5 sampling points. Compared with previous studies, the image acquisition efficiency was greatly improved [15–17], but there were sampling errors. By stitching the images to get complete information of the cotton field, sampling errors can be greatly reduced. But the amount of data is large to process, and can also be affected by the altitude, speed, and spatial resolution of the drone.

Although the proposed method in this study performs well in cotton boll pixel segmentation and yield prediction, there are several aspects that can be further improved and explored. First of all, the images obtained by the drone are orthophotos, and vertical cropping of information may cause some loss. Therefore, we plan to experiment with layered images in the future to reduce information that cannot be displayed because of occlusion. Second, the proposed yield prediction model employs a single input variable and does not consider additional features such as soil type, weather information, and geographic location. Therefore, in future research, more input features will be combined to improve the generalization ability of the model. Overall, this study proposed a new method for yield estimation in densely planted cotton fields based on low-altitude UAV imaging and deep learning, which provides a new idea for cotton yield estimation. Timely and accurate estimation of cotton yield can provide important reference information for cotton producers and agricultural management departments to reasonably determine the storage scale and planting plan, and to assess relevant policies.

## Conclusion

In this work, we proposed and evaluated a cotton yield estimation model, which used DL image processing technology to segment cotton field images acquired by low-altitude UAVs, and the segmented cotton boll pixels ratio was calculated as an input variable for cotton yield estimation. This model could segment cotton boll pixels with a relative error of 0.27–14.35% and an R-square of 0.97, and accurately estimate cotton yield of four fields (38 hectares) with an average error of 6.2%. This study verified the feasibility of estimating cotton yield using low-altitude UAV imaging. The proposed method helps to achieve cotton yield estimation on the field scale while improving the efficiency of cotton yield statistics in Xinjiang. This will provide agricultural scientists, agricultural management departments, and cotton producers with more accurate crop information, enabling them to make scientific decisions. In the future, we will try to apply an approach of layered yield to reduce yield estimation errors in high-density cotton fields.

## Methods

### Data acquisition and experimental platform

**Fig. 3:**
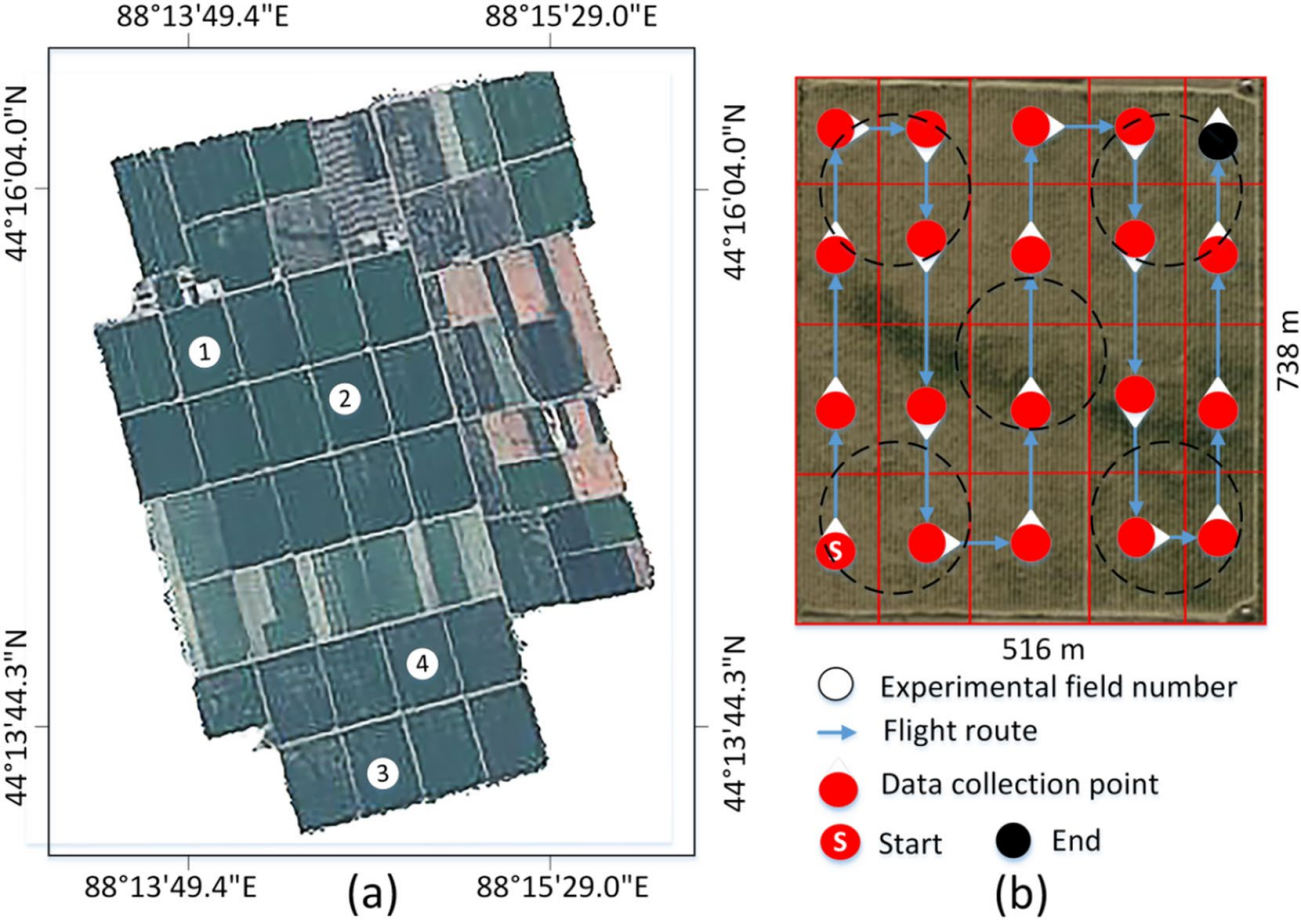
Location of cotton fields. **a** Study area; **b** image acquisition design

**Fig. 4:**
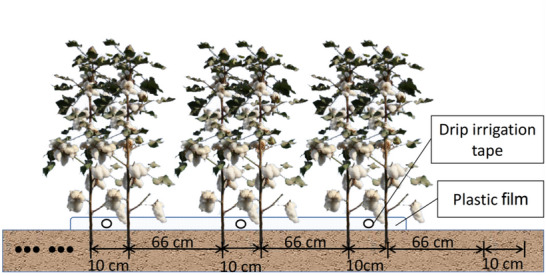
Dense planting pattern of cotton with alternating wide and narrow rows in Xinjiang, China

The experimental fields are located at Tuanjie Farm (44° 13′ 09.3″ N, 88° 16′ 27.3″ E) in Fukang City, Xinjiang Uygur Autonomous Region, China. Four cotton fields (738 m × 516 m) were randomly selected (Fig. 3). The dense planting pattern (alternating wide (66 cm) and narrow (10 cm) rows) (Fig. 4), is widely adopted in Xinjiang, combined with plastic film mulching and drip irrigation. The planting density was 263,000 plants/ha. Field No. 1 was used for model training and testing, and Field No. 2–4 were only used for yield estimation. Images were collected by an industry-grade quadcopter (MATRICE200 V1, DJI Inc., Shenzhen, China) equipped with a cloud platform ZENMUSE X4S and a FC6510 camera. The camera has a fixed focal length of 8.8 mm, F/208-11 focal ratio, and field of view (FOV) of 84°. The image resolution is 5472 × 3078 pixels (JPG format). Data was acquired from October 11 to 18, 2020, after cotton defoliation.

**Fig. 5:**
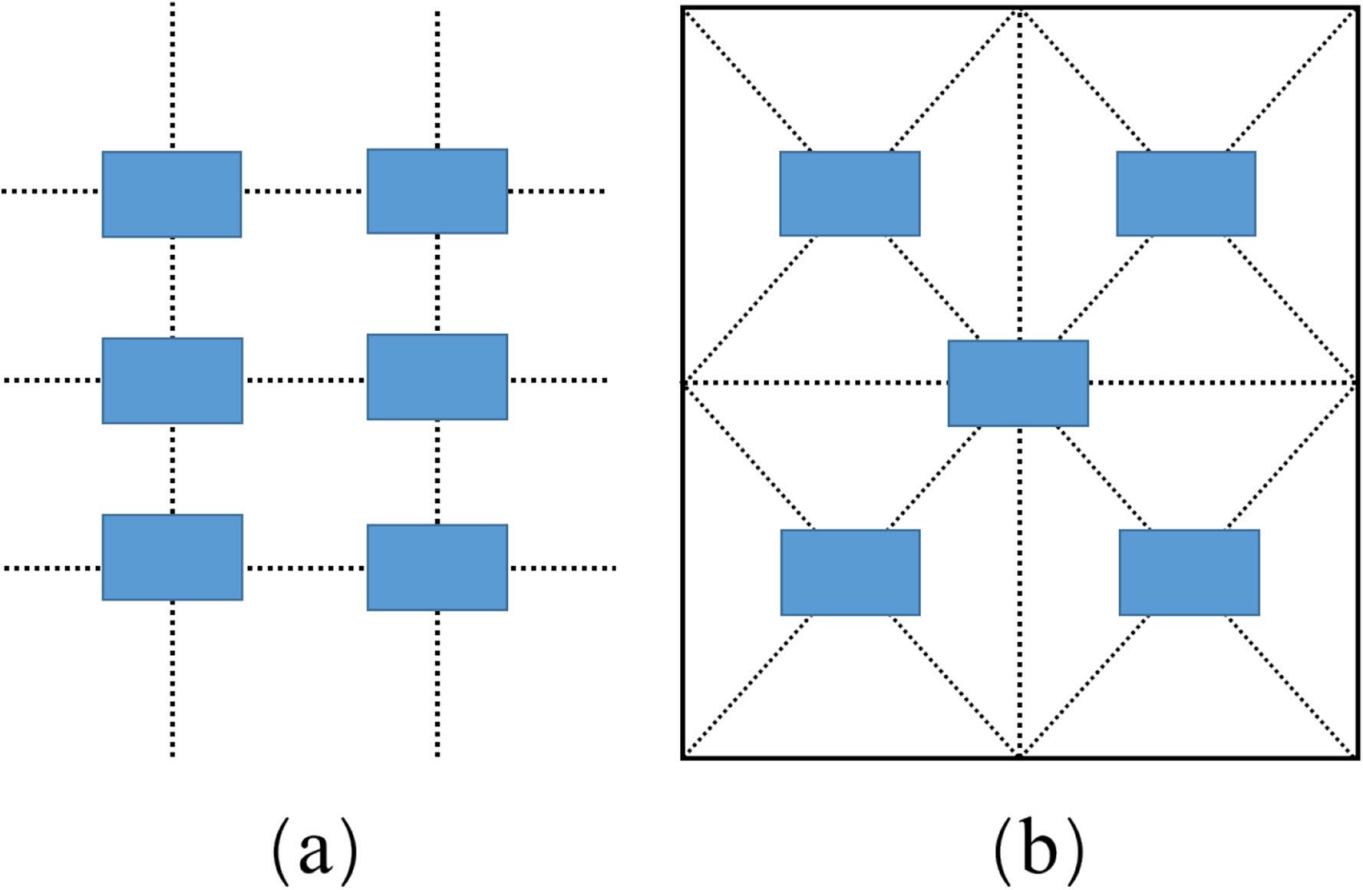
Sampling method. **a** Equidistant sampling method; **b** five-point sampling method

In natural conditions, to maximize the proximity to cotton while avoiding the interference of the UAV rotor airflow to cotton plants, the flying height was set to 5 m. The image resolution was 0.15 cm/pixel. Equidistant sampling method [25] (Fig. 5a) was used to acquire Field No. 1 images along the designed flight route (Fig. 3b), and a sampling area (230 cm × 100 cm) was set at each point. Four coloured flags were used to determine the boundary. To make the images in each sampling area correspond accurately to the yield data, cotton in each sampling area was manually harvested and measured with an electronic scale. The images and yield data for this section are represented in data set 1. Five sampling points [26] (Fig. 5b) were selected to acquire images for Fields No. 1–4. Five images were collected for each cotton field, and a total of 20 images were obtained for yield estimation. Cotton was harvested by a cotton harvester (John Deere CP690, USA) and weighed. The images and yield data for this section are represented in data set 2. The complete data acquisition information is shown in Table 4.

**Table 4:** Data acquisition information

Cotton field	Number of images collected	Methods for image collection	Applications	Yield acquisition method	Data set
1	20	Equidistant sampling method	Model training	Manually harvested and weighed	Data set 1
	5	Five-point sampling method	Yield estimation	Harvested by a cotton harvester and weighed	Data set 2
2	5	Five-point sampling method	Yield estimation	Harvested by a cotton harvester and weighed	Data set 2
3	5	Five-point sampling method	Yield estimation	Harvested by a cotton harvester and weighed	Data set 2
4	5	Five-point sampling method	Yield estimation	Harvested by a cotton harvester and weighed	Data set 2

**Fig. 6:**
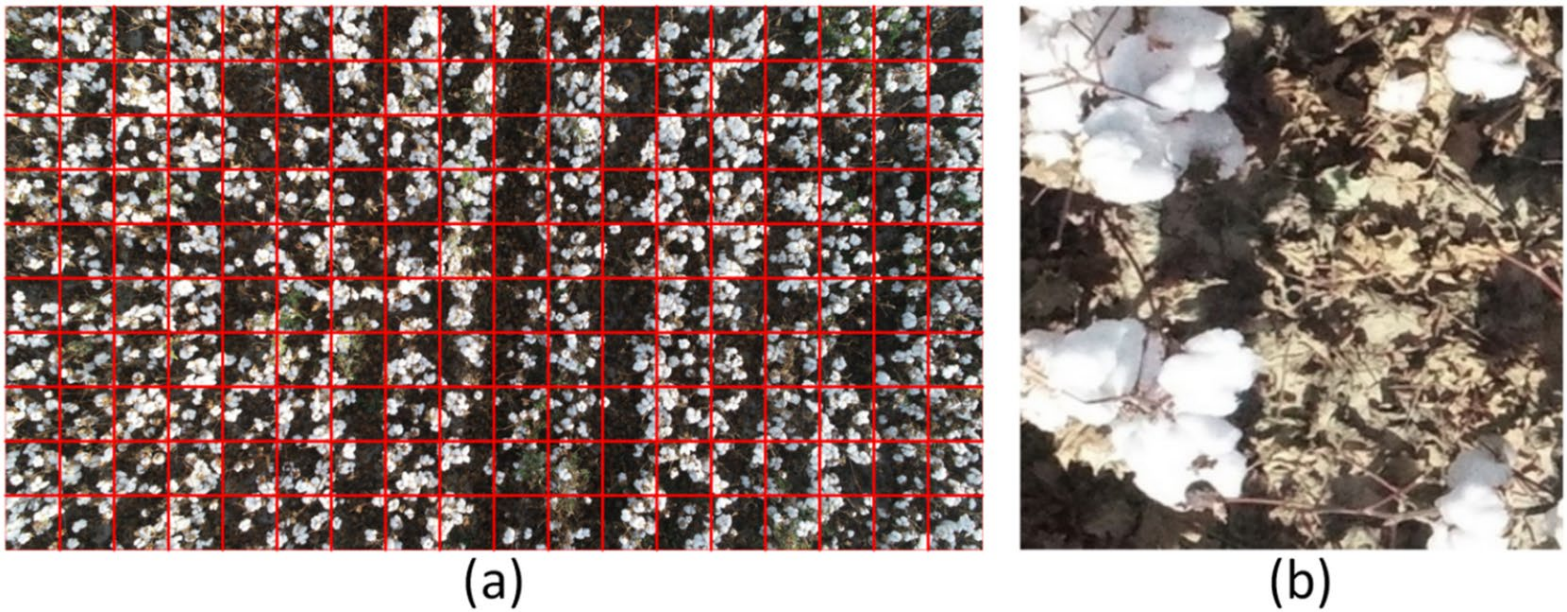
Image cropping. **a** Cropping guide on the original images; **b** cropped image (300 × 300 pixels)

Constrained by computer power, drone images were too large to train deep learning models directly. In this study, the images of data set 1 were processed according to the cropping guide in Fig. 6a, and a total of 4000 sub-images with 300 × 300 pixels were obtained (Fig. 6b). Eighty percent were used as training set and 20% as test set. All image data was tagged interactively using Python's Labelme application. Each image was tagged into two categories: cotton and background, and tagged images were binary images.

### Image feature analysis

**Fig. 7:**
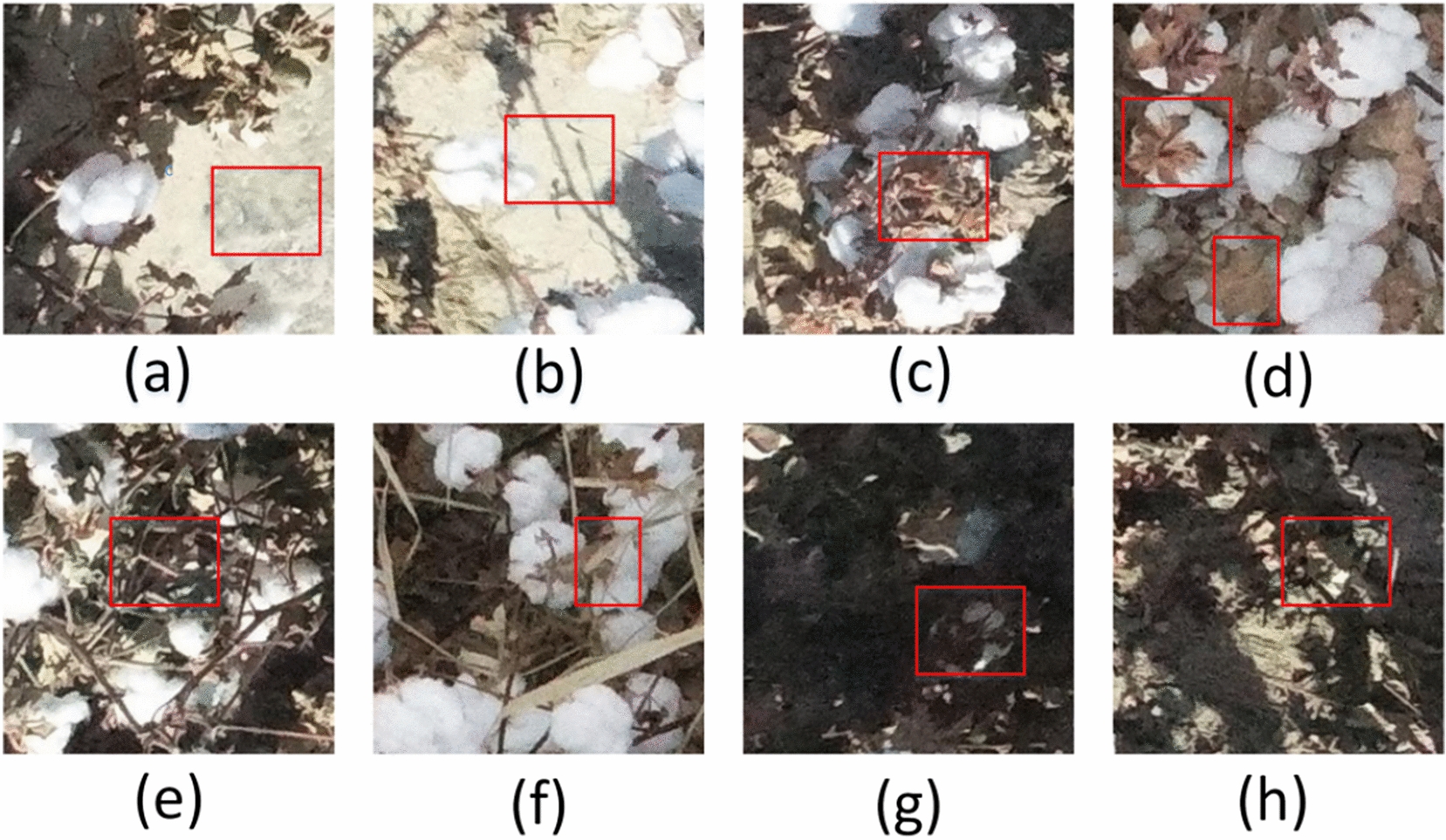
Backgrounds in segmentation: **a** film, **b** soil, **c** cotton leaves, **d** cotton hulls, **e** cotton branches, **f** weeds, **g** cotton bolls in the lower layer, **h** ground

Image segmentation is a specific computer vision task which cannot be simply based on shape, texture, color, and pattern recognition [21]. The background of a cotton field image is complex mainly due to three issues. First, the strong sunlight during the day in Xinjiang overexposes the background of the film (Fig. 7a) and soil (Fig. 7b), which makes cotton bolls look very similar to the background and difficult to be distinguished by a single feature (colour, shape, and texture). Second, backgrounds such as cotton leaves (Fig. 7c), cotton hulls (Fig. 7d), cotton branches (Fig. 7e), and weeds (Fig. 7f) partially occlude cotton bolls, and the occluded area becomes part of the background. Third, orthographic imagery leads to the lower cotton bolls (Fig. 7g) and the ground (Fig. 7h) blocked by the upper layer of cotton plants, resulting in uneven illumination. According to the above analysis, the cotton feature extraction method needs to meet the following requirements:

1. Shallow feature information and high-level semantic information can be extracted simultaneously;
2. Multiscale local information is included;
3. Extracted features are insensitive to changes in light intensity.

Usually, manual extraction of brightness, edges, texture, colour, and other shallow visual features from images cannot meet these requirements very well. Therefore, this study used semantic segmentation to resolve this issue.

### SegNet network architecture

**Fig. 8:**
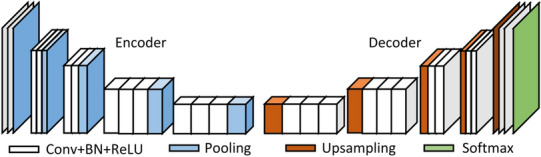
SegNet network

Segnet is a pixel-level semantic segmentation architecture based on convolutional neural network (CNN), which is a symmetrical network composed of an encoder and a decoder [27]. The encoder comprises 5 coding blocks, and each coding block includes a convolutional block and a pooling layer. The convolutional block is composed of a convolutional layer, a batch normalization (BN) layer, and a rectified linear unit (ReLU) layer. Each encoder layer corresponds to a decoder layer. The decoder upsamples the feature images. The upsampled part has more feature channels. The network is used to transfer the context feature information to the higher resolution layer, and ultimately, the feature map size is consistent with the original image size. The Softmax layer is the layer that normalizes the input vector to the probability distribution (Fig. 8).

### CD-SegNet network

**Fig. 9:**
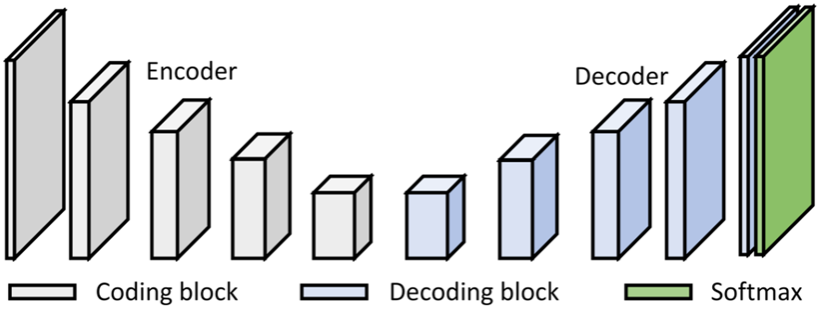
CD-SegNet network

**Fig. 10:**
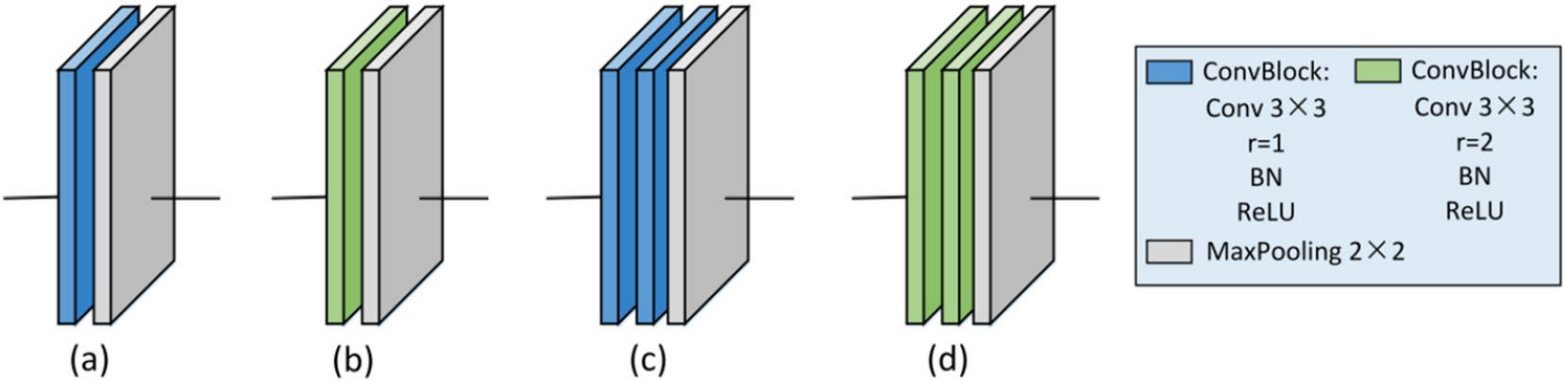
Coding blocks

**Fig. 11:**
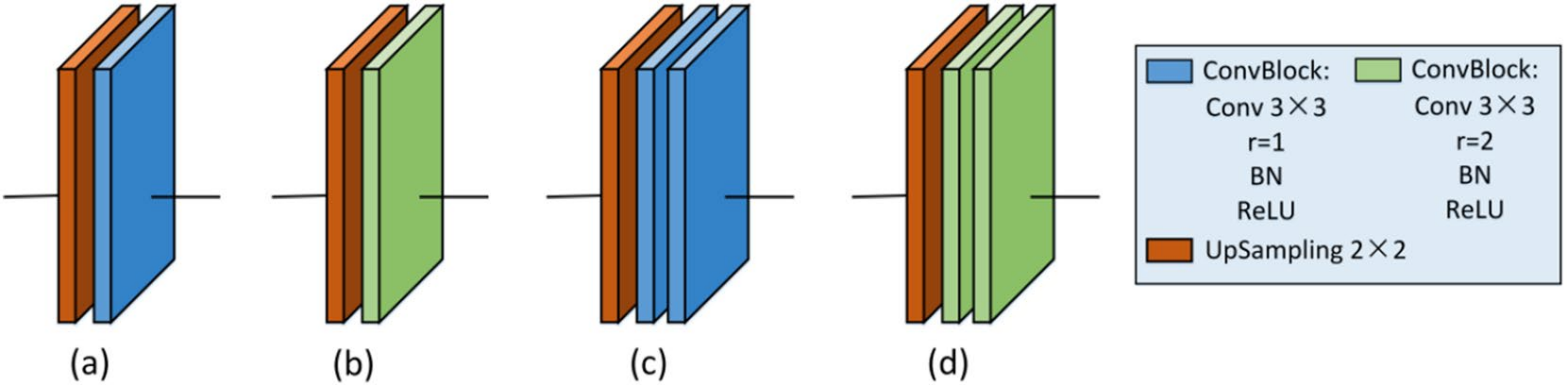
Decoding blocks

The cotton boll dilated convolution SegNet (CD-SegNet) structure used in this study is shown in Fig. 9. The CD-SegNet redesigned and combined the encoding and decoding blocks based on the original SegNet framework. Four new model (Model 1, Model 2, Model 3, and Model 4) were proposed (Table 5). The number of convolutional blocks in the encoding block and decoding block was reduced, and dilated convolution was adopted. The reduction in the number of convolutional blocks could effectively reduce the parameters and improve segmentation efficiency. However, the corresponding receptive field was reduced. Based on a traditional CNN, dilated convolution can expand the feature receptive field without lowering feature spatial resolution. The dilation rate (r) is an important parameter in dilated convolution and represents the degree of expansion [28]. In this model, 3 × 3 convolution blocks were used to replace 7 × 7 and 5 × 5 convolution kernel blocks to save memory (Fig. 10). When r = 1 (Fig. 10a), the receptive field of the input image corresponding to the feature map was 3 × 3 without dilation. When r = 2, the receptive field increased to 5 × 5 (Fig. 10b), and had the same receptive field size (Fig. 10c). However, the number of parameters was reduced by half. For image segmentation, since it is necessary to predict the pixels, the feature map must be upsampled to obtain a feature map with the same size as that of the original image. This process inevitably results in the loss of some information. Therefore, the dilated convolution was added to reduce information loss in this study (Fig. 11).

**Table 5:** Four components of the segmentation model

	Model 1	Model 2	Model 3	Model 4
Coding block	a	b	c	d
Decoding block	a	b	c	d

### Model training

This experiment was conducted using the Windows 10 desktop operating system on an Intel(R) Gold6126 CPU processor, with a default frequency of 2.60 GHZ and memory of 64 GB. The graphics card used was an NVIDIA GeForce RTXTM2060 (with 6G video memory), and the Python version was 3.6, compiled on Jupyter in Anaconda. The Pytorch was used as the DL framework, and a combination of Cuda 10.0 and cudnn 7.4.1.5 was used for GPU acceleration to improve the model training speed. The model gradient descent adopted the adaptive momentum stochastic optimization method (Adam). The learning rate was 0.001, and the beta first-order and second-order attenuation coefficients were set to 0.9 and 0.98, respectively. The training-related parameters are shown in Table 6.

**Table 6:** Training-related parameters of deep learning segmentation model

GSD	Image size	Epoch	Learning rate	Batch size	Sample number of training set	Sample number of validation set
0.15	300 × 300	50	0.001	64	3200	800

### Evaluation metrics

In this study, the pixel accuracy (PA), recall, mean intersection over union (MIOU), and F1 score (F1-score) were used to evaluate the segmentation accuracy. All the evaluation indicators used were calculated from the parameters in the confusion matrix (Table 7). In the model accuracy evaluation, the confusion matrix was mainly used to compare predicted values with measured values, and was calculated by comparing the position of each measured pixel with the position of the predicted pixel.

**Table 7:** Confusion matrix

Confusion matrix		True value	
		Positive	Negative
Predicted value	Positive	True positive (TP)	False positive (FP)
	Negative	False negative (FN)	True negative (TN)

PA refers to the ratio of correctly classified pixels in the total pixels (Eq. 2). Recall score represents the model’s ability to correctly predict the positives out of actual positives (Eq. 4). The intersection ratio (intersection over union, IOU) is a standard metric used to evaluate the accuracy of semantic segmentation (Eq. 1). MIOU refers to the average of all categories of IOU (Eq. 5). F1-score is the harmonic mean of the precision and recall, which is used in statistics and as indicator to measure the accuracy of a binary classification (Eq. 3).

1 $$\begin{array}{c}IOU=\frac{TP}{TP+FP+FN}\end{array}$$

2 $$\begin{array}{c}CPA=\frac{TP}{TP+FP}\end{array}$$

3 $$\begin{array}{c}F1 Score=\frac{2CPA*Recall}{CPA+Recall}\end{array}$$

4 $$\begin{array}{c}Recall=\frac{TP}{TP+FN}\end{array}$$

5 $$\begin{array}{c}MIOU=\frac{1}{k+1}\sum_{i=0}^{k}\frac{TP}{TP+FP+FN}\end{array}$$

where TP and TN stand for the number of pixels correctly classified for the cotton and non-cotton classes, and FP and FN stand for the number of misclassified pixels.

Coefficient of determination (R^2^) was used to quantify the accuracy of model segmentation.

6 $$\begin{array}{c}{R}^{2}=1-\frac{{\sum }_{i=1}^{n}{({t}_{i}-{c}_{i})}^{2}}{{\sum }_{i=1}^{n}{({t}_{i}-\overline{{t }_{i}})}^{2}}\#\end{array}$$

where ${\mathrm{t}}_{\mathrm{i}}$ and ${\mathrm{c}}_{\mathrm{i}}$ are the number of pixels of cotton bolls segmented by the model and the measured number of pixels of cotton bolls in the image, respectively, and $\overline{{\mathrm{t}}_{\mathrm{i}}}$ is the average value of the measured number of pixels of cotton bolls in the image.

### Yield estimation analysis

Regression analysis is a statistical analysis method for determining the interdependent quantitative relationship between two or more variables. Linear regression is one of the most widely used regression analysis methods, and it is also the preferred regression analysis method. In this study, linear regression analysis model with SciPy computing library was used to analyse the relationship between cotton boll pixels ratio in the sampling area and the measured yield of the sampling area. A total of 20 samples were used. Once the regression model was obtained, the yield per hectare of the cotton field was calculated through the regression model, and the relative error was calculated by comparing with measured yield. The cotton boll pixels ratio of the whole cotton field was the average of five images in data set 2. Equation 7 was used for calculating the pixels ratio of cotton bolls.

7 $${\text{Cotton boll pixels ratio}} = \frac{{\text{Number of cotton boll pixels in the image}}}{{\text{Total number of pixels in the image}}}$$

## Data Availability

The datasets used and/or analysed during the current study are available from the corresponding author on reasonable request.

## References

[1] Deng Y, Ning S (2020). The status quo of the development of machine-picked cotton in Xinjiang and the solutions and prospects for several issues. Cotton Sci.

[2] Cao W, Liu J, Zhao L (2007). Research on optimal time selection for cotton yield estimation by remote sensing in northern Xinjiang. China Cotton.

[3] Meng L, Zhang X-L, Liu H (2017). Estimation of cotton yield using the reconstructed time-series vegetation index of landsat data. Can J Remote Sens.

[4] He L, Mostovoy G (2019). Cotton yield estimate using sentinel-2 data and an ecosystem model over the southern US. Remote Sens.

[5] Dalezios NR, Domenikiotis C, Loukas A (2001). Cotton yield estimation based on NOAA/AVHRR produced NDVI. Phys Chem Earth Part B Hydrol Oceans Atmos.

[6] Meng L, Liu H, Zhang X (2019). Assessment of the effectiveness of spatiotemporal fusion of multi-source satellite images for cotton yield estimation. Comput Electron Agric.

[7] Duan T, Chapman SC, Guo Y (2017). Dynamic monitoring of NDVI in wheat agronomy and breeding trials using an unmanned aerial vehicle. Field Crop Res.

[8] Jin X, Liu S, Baret F (2017). Estimates of plant density of wheat crops at emergence from very low altitude UAV imagery. Remote Sens Environ.

[9] Cuaran J, Leon J (2021). Crop monitoring using unmanned aerial vehicles: a review. Agric Rev.

[10] Turner D, Lucieer A, Malenovsky Z (2014). Spatial co-registration of ultra-high resolution visible, multispectral and thermal images acquired with a Micro-UAV over Antarctic Moss Beds. Remote Sens.

[11] Zhang C, Kovacs JM (2012). The application of small unmanned aerial systems for precision agriculture: a review. Precision Agric.

[12] Ashapure A, Jung J, Chang A (2020). Developing a machine learning based cotton yield estimation framework using multi-temporal UAS data. ISPRS J Photogramm Remote Sens.

[13] Stroppiana D, Migliazzi M, Chiarabini V, et al. Rice yield estimation using multispectral data from UAV: A preliminary experiment in northern Italy [C]. IEEE International Geoscience and Remote Sensing Symposium (IGARSS), 2015: 4664–4667.

[14] Huang Y, Brand HJ, Sui R (2016). Cotton yield estimation using very high-resolution digital images acquired with a low-cost small unmanned aerial vehicle. Trans ASABE.

[15] Feng A, Sudduth K, Vories E, et al. Cotton yield estimation based on plant height from UAV-based imagery data. 2018 ASABE Annual International Meeting, 2018: 1.

[16] Feng A, Zhou J, Vories ED (2020). Yield estimation in cotton using UAV-based multi-sensor imagery. Biosys Eng.

[17] Xu W, Chen P, Zhan Y (2021). Cotton yield estimation model based on machine learning using time series UAV remote sensing data. Int J Appl Earth Obs Geoinform.

[18] Xu W, Yang W, Chen S (2020). Establishing a model to predict the single boll weight of cotton in northern Xinjiang by using high resolution UAV remote sensing data. Comput Electron Agric.

[19] Dube N, Bryant B, Sari-Sarraf H (2020). Cotton boll distribution and yield estimation using three-dimensional point cloud data. Agron J.

[20] Sun S, Li C, Paterson AH (2019). Image processing algorithms for infield single cotton boll counting and yield prediction. Comput Electron Agric.

[21] Li Y, Cao Z, Xiao Y (2017). DeepCotton: in-field cotton segmentation using deep fully convolutional network. J Electron Imaging.

[22] Ma J, Li Y, Liu H (2020). Improving segmentation accuracy for ears of winter wheat at flowering stage by semantic segmentation. Comput Electron Agric.

[23] Singh N, Tewari V, Biswas P (2021). Image processing algorithms for in-field cotton boll detection in natural lighting conditions. Artif Intell Agric.

[24] Singh N, Tewari V, Biswas P, et al. Semantic segmentation of in-field cotton bolls from the sky using deep convolutional neural networks. Smart Agric Technol. 2022: 100045.

[25] Zhao H-S, Zhu X-C, Li C (2017). Improving the accuracy of the hyperspectral model for apple canopy water content prediction using the equidistant sampling method. Sci Rep.

[26] Jin X, Yang G, Xu X (2015). Combined multi-temporal optical and radar parameters for estimating lai and biomass in winter wheat using HJ and Radarsar-2 data. Remote Sens.

[27] Badrinarayanan V, Kendall A, Cipolla R (2017). SegNet: a deep convolutional encoder-decoder architecture for image segmentation. IEEE Trans Pattern Anal Mach Intell.

[28] Yu F, Koltun V. Multi-scale context aggregation by dilated convolutions. arXiv preprint arXiv:1511.07122, 2015.

